# Evaluating and optimising performance of multi‐species call recognisers for ecoacoustic restoration monitoring

**DOI:** 10.1002/ece3.10309

**Published:** 2023-08-22

**Authors:** Simon Linke, Daniella Teixeira, Katie Turlington

**Affiliations:** ^1^ CSIRO Environment Dutton Park Queensland Australia; ^2^ Australian Rivers Institute Griffith University Nathan Queensland Australia; ^3^ School of Biology and Environmental Science Queensland University of Technology Brisbane Queensland Australia; ^4^ Bush Heritage Australia Melbourne Victoria Australia

## Abstract

Monitoring the effect of ecosystem restoration can be difficult and time‐consuming. Autonomous sensors, such as acoustic recorders, can aid monitoring across long time scales. This project successfully developed, tested and implemented call recognisers for eight species of frog in the Murray–Darling Basin. Recognisers for all but one species performed well and substantially better than many species recognisers reported in the literature. We achieved this through a comprehensive development phase, which carefully considered and refined the representativeness of training data, as well as the construction (amplitude cut‐off) and the similarity thresholds (score cut‐offs) of each call template used. Recogniser performance was high for almost all species examined. Recognisers for *Crinia signifera*, *Limnodynastes fletcherii*, *Limnodynastes dumerilii*, *Litoria peronii* and *Crinia parinsignifera* all performed well, with most templates having receiver operating characteristics values (the proportion of true positive and true negatives) over 0.7, and some much higher. Recognisers for *L. peronii*, *L. fletcherii* and *L. dumerilii* performed particularly well in the training data set, which allowed for responses to environmental watering events, a restoration activity, to be clearly observed. While slightly more involved than building recognisers using commercial packages, the workflows ensure that a high‐quality recogniser can be built and the performance fine‐tuned using multiple parameters. Using the same framework, recognisers can be improved on in future iterations. We believe that multi‐species recognisers are a highly effective and precise way to detect the effects of ecosystem restoration.

## Graphical abstract

This project successfully developed, tested and implemented a workflow to develop call recognisers for eight species of frog in the Murray–Darling Basin. Recognisers for all but one species performed well and substantially better than many species recognisers reported in the literature.

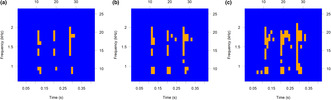

## 1 INTRODUCTION

Bioacoustics—the study of sound production, dispersion and reception in animals—has been practiced for millennia. Even in underwater systems, Aristotle described communication between animals in great anatomic and behavioural detail (Aristotle, 1910; Linke et al., 2020). Bioacoustics can be used to study animal ecology—for example, reproductive behaviour and success (Teixeira et al., 2019)—to monitor population dynamics of native or invasive species (Brodie, Yasumiba, Towsey, Roe, & Schwarzkopf, 2020) and to detect rare and endangered soniferous animals (Dema et al., 2020; Dutilleux & Curé, 2020; Znidersic et al., 2020). The sister discipline ecoacoustics is a new field that is not restricted to biotic organisms, but—like ecology to biology—investigates acoustic diversity, and its relation to habitats as well as populations and ecological communities (Sueur & Farina, 2015).

Ecoacoustics has been used to quantify ecological responses to environmental restoration or improvement in condition, providing a rapid and continuous monitoring framework that can detect both degradation and restoration success (Greenhalgh et al., 2021; Linke & Deretic, 2020; Znidersic & Watson, 2022). Often, acoustic indices are used in assessments. These indices are analogous to measurements of diversity or richness in classical ecology—they summarise the acoustic properties of an overall soundscape, for example its spatial, temporal or combined complexity, its overall volume or the relation between natural and human‐influenced frequency bands (Buxton et al., 2018; Sueur et al., 2014). However, given inherent variations in soundscapes between places, ecoacoustic indices must be calibrated by the ecosystem (Bradfer‐Lawrence et al., 2019; Fairbrass et al., 2017). While some authors have described clear variation along landscape gradients (Ng et al., 2018), others have found little relation between acoustic indices to human disturbance (Mitchell et al., 2020). Other studies have found that acoustic indices can be dominated by single acoustic events, for example river flows (Linke & Deretic, 2020) or single species that dominate the soundscape, such as snapping shrimp (Bohnenstiehl et al., 2018).

Call recognisers usually function to detect single species, since bioacoustics is often used to detect cryptic or rare animals (Leseberg et al., 2020). However, as the application of acoustics to environmental monitoring increases, multi‐species recognisers are likely to become more important. Multi‐species recognisers detect sympatric species simultaneously (Wright et al., 2020; Zhong et al., 2020), and outputs can be analysed for species separately or combined. This is useful where groups of species (e.g. mixed species frog choruses) represent environmental change or other ecological values. Like single‐species recognisers, multi‐species recognisers can use acoustic indices to detect soundscapes in which target species are likely to occur (Brodie, Allen‐Ankins, et al., 2020), or they can implement several single‐species algorithms to detect discrete calls (Ruff et al., 2020). There are many challenges to creating reliable multi‐species recognisers, however, methods for reducing the increased risk of false detections are beginning to be examined (Campos et al., 2019; Wright et al., 2020).

Performance metrics used to evaluate and report on‐call recogniser performance are highly variable in the literature (Knight et al., 2017). This makes comparisons and repeatability difficult. Perhaps more importantly, there are major inconsistencies in the type and amount of training data used and the test data sets upon which recognisers are evaluated. For example, test data sets can vary between single calls and complex soundscapes—the latter of course being a lot more error‐prone. While strictly standardised methods are unlikely to be feasible (e.g. for rare species, data sets can be extremely difficult to acquire), studies should, as a minimum, report the representativeness of the training data, how these were chosen or tested, and any limitations or assumptions. Moreover, the extent to which training and test data include real‐world ambient noise should be explained, because factors like wind, noise and other species' calls can significantly impact false detections (Crump & Houlahan, 2017; Kahl et al., 2021; Priyadarshani et al., 2018; Salamon et al., 2016; Towsey et al., 2012). To standardise the reporting of performance metrics, Knight et al. (2017) recommended all studies report precision, recall, *F*‐score and area under the precision‐recall curve (AUC) or, for comparison with the broader classifier literature, receiver operating characteristics (ROC) curve. In this study, we propose an evaluation and calibration system using the metrics proposed by Knight et al. (2017) embedded in a ‘real world’ testing environment. While many other studies evaluate the success of their recognisers on isolated calls, our system tests on similar soundscapes that the recognisers will be run on, which include similar sounds, weather events or other signals that can ‘confuse’ a recogniser.

We are using frog responses to ecosystem restoration as our case study to develop multi‐species acoustic recognisers. Linke and Deretic (2020) pioneered the use of ecoacoustic analysis as a tool to continuously monitor populations after restorative water returns to wetlands. When manually listening to recordings of frog and bird calls, they found highly significant responses in the richness of water‐dependent biota to environmental watering. However, the response of acoustic indices was much weaker, and in some cases, non‐significant, partially obfuscated by ambient noises, and also subject to high diurnal variation. This led the authors to conclude that a logical next step was to trial multi‐species call recognisers that would combine the advantage of species specificity with the automated processing of acoustic indices (Linke & Deretic, 2020).

Using a template‐matching algorithm (binary point matching, Towsey et al., 2012) in the R package monitoR (Katz et al., 2016b), we aimed to establish a free and open source protocol to optimise multi‐species call recogniser construction and evaluation using three levers: template selection, amplitude cut‐off and score cut‐off.

- First, we tested the performance of geographically representative candidate call templates (training data) and, from this, selected a small number of high‐performing templates from which to construct call recognisers.
- Second, we examined call templates at a range of amplitude cut‐offs, which alters their detection sensitivity.
- Third, we tested templates across a wide range of score cut‐offs, which defines the threshold of similarity between templates and sound data at which detection is returned.

As a case study, we tested this protocol on the calls of eight sympatric frog species from the Koondrook‐Pericoota wetland complex in the Murray–Darling Basin, Australia.

## 2 METHODS

### 2.1 Overview and computational strategy

**FIGURE 1:**
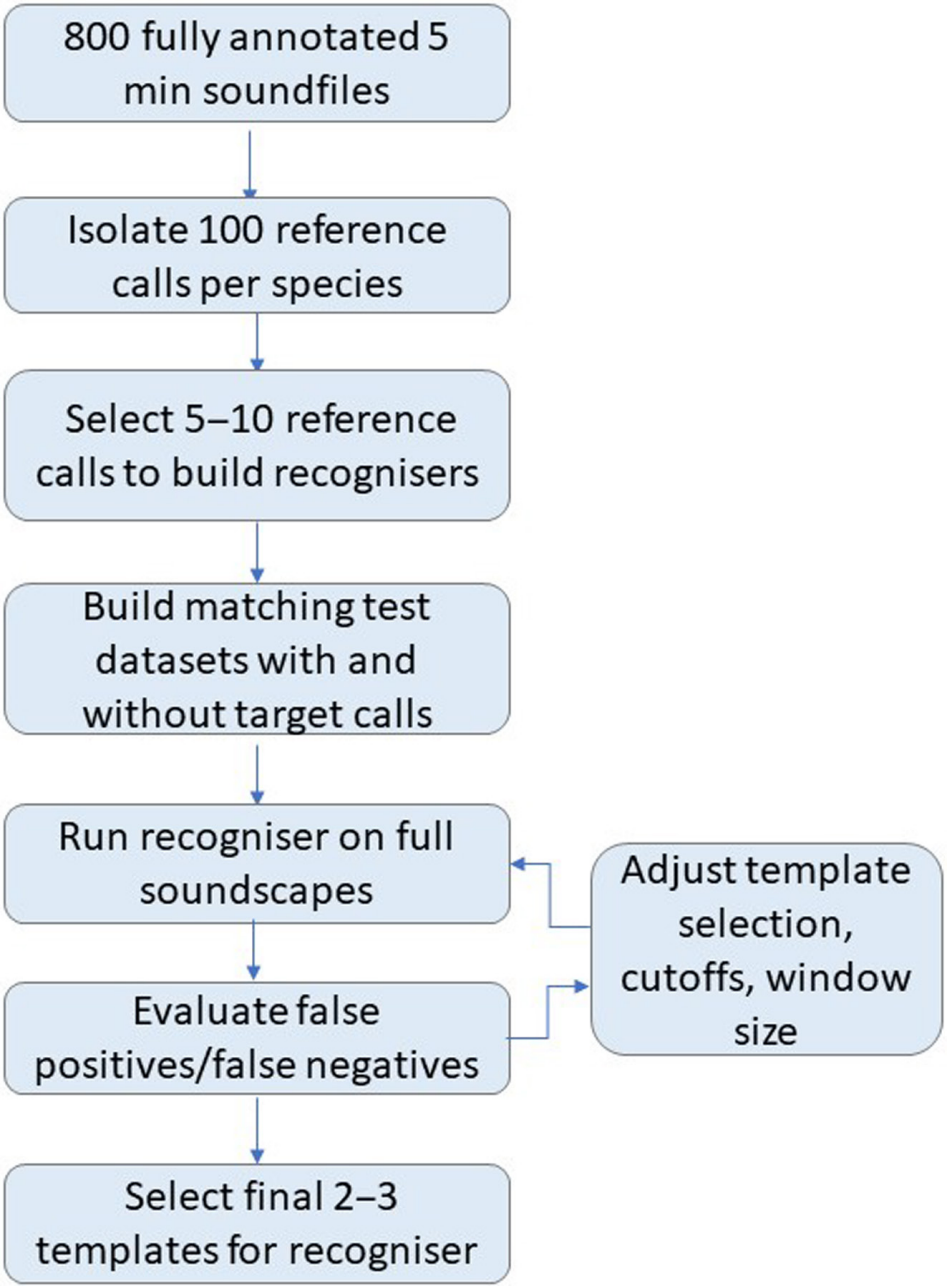
Flow chart for recogniser construction.

To build and evaluate the recognisers for eight target frog species, we used a large database of annotated calls from the study area in Koondrook‐Perricoota (KP) forest—an extensive forest of river red gums along the Murray river near the towns of Deniliquin and Echuca (Appendix 1). The Forestry Corporation of the Australian state of New South Wales—the body commissioning the study—had previously annotated 831 files from 20 sites and found varying levels of presence for the different species. From these files, we extracted between 100 and 200 reference calls per species, from all sites where the species was detected (Figure 1). Following manual inspections for call clarity and variation, we used these reference calls to build approximately 5–10 candidate recognisers per species (i.e. one template equals one recogniser per species). Some recognisers were based on templates of the same reference call but were created using different amplification settings, which is modifiable in monitoR. We then ran the recognisers on the pre‐annotated files to calibrate the score cut‐off (similarity threshold between reference call templates and the sound files) and estimate omission and commission errors. We then chose the final recognisers based on the best Receiver Operator Criterion (ROC, Zou et al., 2007), thus minimising both false positive and false negative detections.

### 2.2 Study area and data collection

The Forestry Corporation of NSW provided 2 years of acoustic data files recorded between February 2018 and February 2020, before and after environmental watering events. A SongMeter 3 or SongMeter 4 (Wildlife Acoustics Inc.) sound recorder was deployed at each of the 20 study sites (see Appendix 1) in the KP Forest. Recorders were set to 44 kHz. Prior to January 2019, each recorder recorded 5 min of audio per hour. After January 2019, this was changed to 1 min per hour. From the acoustic data provided by the Forestry Corporation of NSW, eight frog species were identified as potential indicators of ecological health. A list of previously annotated detections (i.e. times, dates and locations that these frogs had been detected via manual listening) was also provided by the Forestry Corporation of NSW (see Table 1 for the number of files where the candidate frog species were present in annotated files).

**TABLE 1:** Count of annotated evaluation files where calls of candidate frog species were present.

Species	Evaluation files (*n*)
*Crinia signifera*	300
*Limnodynastes tasmaniensis*	146
*Limnodynastes fletcherii*	78
*Limnodynastes dumerilii*	74
*Litoria peronii*	118
*Crinia parinsignifera*	298
*Neobatrachus sudelli*	62
*Litoria raniformis*	24

To build a training data set of calls for recogniser development, we first manually selected and extracted a minimum of 100 reference calls for each species using Adobe Audition CC and Raven Pro 1.5 software (spectrograms using a Hamming window at 1024 samples). To maximise representativeness, we (a) selected calls from as many survey sites as possible, to capture geographical variation and (b) selected calls of varying quality and amplitude, to capture soundscape variation. These are important steps to improve the similarity between call templates and ‘real‐world’ sound data. Building a recogniser solely from calls that are loud and clear would perform poorly if the species' calls are rarely loud and clear in field recordings. Given the complexity of frog choruses, variations in ambient noise and differences in amplitude among calls (e.g. from variations in the distance of the frog from the sound recorder), capturing diversity in call templates is a critical component of recogniser construction.

### 2.3 Recogniser construction

**FIGURE 2:**
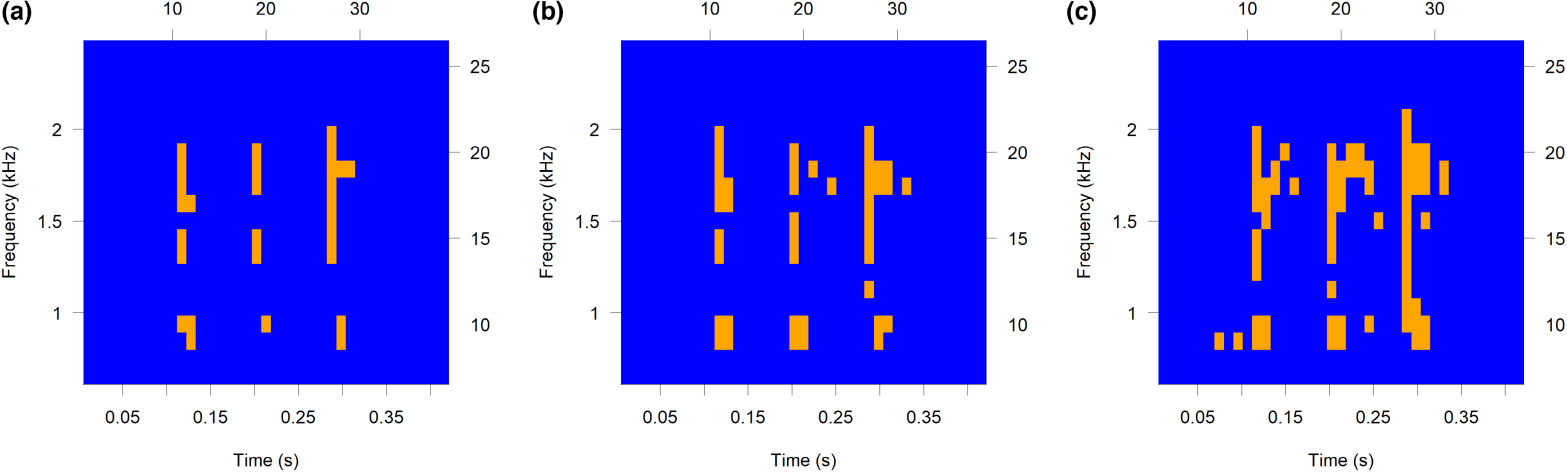
Example of amplitude cut‐offs of (a) −31 dB, (b) −34 dB, (c) −37 dB for a single *Limnodynastes tasmaniensis* template. Orange indicates ‘on’ (call) regions and blue indicates ‘off’ (non‐call) regions.

To construct the recognisers, we used the technique ‘binary template matching’—a technique that first converts a spectrogram into a binary template and then matches ‘on’ and ‘off’ points of the template to the file the recogniser is applied to Katz et al. (2016b) and Towsey et al. (2012). This was done using the monitoR package in R, which provides flexibility in its construction parameters (Katz et al., 2016b). To build the initial recognisers, templates were constructed from a minimum of 10 reference calls (from the pool of 100 candidate calls) that were both clear and representative of the variation in calls and environmental conditions. Binary templates comprise ‘on’ and ‘off’ regions (call and non‐call), which are based on a user‐defined amplitude cut‐off (Figure 2). Each template's amplitude cut‐off was determined through manual inspection of templates using the makeBinTemplate function in monitoR (Katz et al., 2016b). Amplitude cut‐offs were set arbitrarily and progressively altered and reviewed. A cut‐off that clearly showed call structure and was not masked by background noise, was deemed appropriate, with some background noise deemed acceptable.

The lower and upper bounds of the template's frequency limits were manually chosen to capture as much of the call as possible, while minimising potential overlap with common noise sources (e.g. crickets). Most templates were constructed with a window size of 512 samples. Templates for *Crinia signifera* used a window size of 256 samples to improve the resolution of the species' highly pulsatile call. Both the frequency limit and the window size affect the number of on and off points in the template and, therefore, the processing speed of a template. For *Limnodynastes tasmaniensis*, we trialled templates with both window sizes but chose to use the 512 sample templates as they showed the call's structure more clearly.

### 2.4 Recogniser evaluation and score cut‐off

**FIGURE 3:**
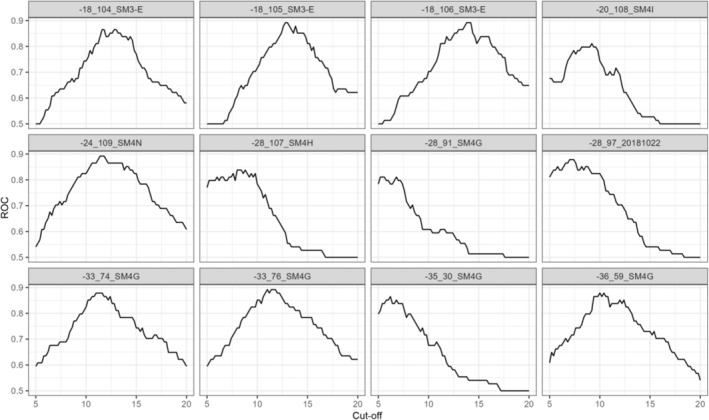
Receiver operating characteristics (ROC) plots for different templates to detect *Limnodynastes dumerili*. Templates 3, 8 and 11 were chosen with the score cut‐off corresponding to the highest ROC.

For each template, we tested a range of score cut‐offs, which is a user‐defined similarity threshold at which a template will return a detection. This threshold alters the proportion of true positive and false positive detections and is, therefore, an important part of optimising call recognisers (Katz et al., 2016a). For each species, we tested the recognisers at a low score cut‐off of 3; thus, any call instance that scored 3 or higher was returned as a detection by monitoR. Optimal score cut‐off for each template was determined by constructing receiver operating characteristic (ROC) curves, a diagnostic tool that optimises that trade‐off between false positive and true positive rates (Figure 3). We calculated true‐ and false positive rates at score cut‐off increments of 0.2 and determined the optimum as the score cut‐off where true positives were greatest relative to false positives (i.e. the peaks in Figure 3). We then retained these score cut‐offs for the recogniser evaluation.

### 2.5 Recogniser evaluation and detection of false positives

Recogniser performance was evaluated on a manually verified and balanced subsample of 1‐min sound recordings that were categorised for each species' presence or absence. The sample size of evaluation files varied among species. Any detection returned in a recording where the species was present was taken to be a true positive detection. All other detections were deemed false positives. For each template, we quantified the number of call detections in sound files where the species was present (true positive count; Count TP) and absent (false positive count; Count FP) and the number of sound files in which the presence/absence (PA) of species was correctly detected (true presence: PA TP), incorrectly detected (false presence; PA FP), missed (false absence; PA FN) or correctly undetected (true absence; PA TN). Using these values, for each template, we calculated precision, recall and ROC value, which are given by the formulas:

$$\text{Precision}=\text{True Positives}/\left(\text{True Positives}+\text{False Positives}\right)$$

$$\text{Recall}=\text{True Positives}/\left(\text{True Positives}+\text{False Negatives}\right)$$

$$\mathrm{ROC}=\left(\text{True Positives}+\text{True Negatives}\right)/\text{Number of evaluation files}$$

Sample detections of false positives were manually verified. To determine the source of false positive, files that the recognisers deemed as having the frog species present were cross‐examined against the manual detection list provided by the Forestry Corporation. This presented a list of unverified false positive detections—where the recognisers identified a call, but the original manual detection list indicated the frog species was not present. A subsample of these false positive detections was manually verified through audio and visual cues on Adobe Audition CC. The false positive detection files were reviewed until the source categories of these false positive detections were considered consistent.

## 3 RESULTS

### 3.1 Recogniser performance evaluation

Recognisers performed well for most species (Table 3). Performance was high (ROC > 0.8) for templates of *L. dumerilii*, *L. fletcheri* and *L. peronii*. Performance was also high for *N. sudelli* and *L. raniformis* but the sample sizes of their evaluation files were relatively small (Table 2). Performance was moderately high (ROC > 0.7) for most templates of *C. signifera* and *C. parinsignifera*. Conversely, performance was poor for *L. tasmaniensis* (ROC < 0.6), for which most templates showed low precision and moderate recall.

**TABLE 2:** Performance evaluation of final recognisers.

Species	Template name	Count FP	Count TP	Call precision	PA FP	PA TP	PA FN	PA TN	Survey precision	Survey recall	ROC
*Crinia signifera*	‐43_129_SM4S	1048	7963	0.884	28	116	34	122	0.806	0.773	0.793
*Crinia signifera*	‐46_130_SM4S^a^	4432	18,488	0.807	41	127	23	109	0.756	0.847	0.787
*Crinia signifera*	‐47_183_SM4H	2211	18,609	0.894	40	120	30	110	0.750	0.800	0.767
*Limnodynastes tasmaniensis*	512 5khz_‐27_95_SM3‐E+0	10,509	4245	0.288	37	46	27	36	0.554	0.630	0.562
*Limnodynastes tasmaniensis*	512 5khz_‐32_45_SM4G^a^	18,671	9747	0.343	46	60	13	27	0.566	0.822	0.596
*Limnodynastes tasmaniensis*	512 5khz_‐32_90_SM3‐E+0	1807	1134	0.386	25	37	36	48	0.597	0.507	0.582
*Limnodynastes fletcherii*	‐23_95_SM3‐E	52	9438	0.995	7	36	3	32	0.837	0.923	0.872
*Limnodynastes fletcherii*	‐25_29_SM4G	2	1041	0.998	1	27	12	38	0.964	0.692	0.833
*Limnodynastes fletcherii*	‐31_54_SM4G	381	6531	0.945	6	37	2	33	0.860	0.949	0.897
*Limnodynastes fletcherii*	‐34_3_SM3‐E^a^	903	1866	0.674	14	38	1	25	0.731	0.974	0.808
*Limnodynastes dumerilii*	‐18_104_SM3‐E^a^	47	3253	0.986	11	35	2	26	0.761	0.946	0.824
*Limnodynastes dumerilii*	‐18_106_SM3‐E	7	1964	0.996	4	32	5	33	0.889	0.865	0.878
*Limnodynastes dumerilii*	‐28_97_20181022 (SM4G)	3	4043	0.999	2	33	4	35	0.943	0.892	0.919
*Limnodynastes dumerilii*	‐35_30_SM4G	19	3452	0.995	5	36	1	32	0.878	0.973	0.919
*Litoria peronii*	‐30_256_60_SM3‐E	31	1874	0.984	9	45	14	50	0.833	0.763	0.805
*Litoria peronii*	‐31_512_65_SM3‐E	9	806	0.989	5	46	13	54	0.902	0.780	0.847
*Litoria peronii*	‐38_256_75_SM3‐E	2	678	0.997	1	42	17	58	0.977	0.712	0.847
*Crinia parinsignifera*	‐30_37_SM4S	1273	6201	0.830	33	100	49	116	0.752	0.671	0.725
*Crinia parinsignifera*	‐33_38_SM4S	1520	4563	0.750	26	91	58	123	0.778	0.611	0.718
*Crinia parinsignifera*	‐41_47_SM3‐E0+1^a^	6707	10,575	0.612	58	109	40	91	0.653	0.732	0.671
*Neobatrachus sudelli*	‐23_512_94_SM4M	105	3032	0.967	1	31	0	30	0.969	1.000	0.984
*Neobatrachus sudelli*	‐39_512_100_SM4M	36	2645	0.987	1	31	0	30	0.969	1.000	0.984
*Neobatrachus sudelli*	‐52_512_1_SM4M	3	2097	0.999	1	31	0	30	0.969	1.000	0.984
*Litoria raniformis*	‐36_512_24_SM4R	0	28	1.000	0	10	2	12	1.000	0.833	0.917
*Litoria raniformis*	‐42_512_85_SM4R	8	962	0.992	2	12	0	10	0.857	1.000	0.917
*Litoria raniformis*	‐44_512_99_SM4R	0	100	1.000	0	10	2	12	1.000	0.833	0.917

*Note*: Counts are the count of call detections in sound files, where the species was absent (Count FP) or present (Count TP). Presence–absence (PA) values are the number of sound files, where the species was missed (PA FN), correctly detected (PA TP), incorrectly detected (PA FP) or correctly undetected (PA TN). Performance metrics are precision (TP/TP + FP) for calls (Call Precision) and PA in files (Survey Precision), recall (TP/TP + FN) for PA in files and ROC.

^a^ Template excluded from final analyses.

The *L. fletcherii* recogniser comprised two templates from two sites. All templates performed well. The first template had very high precision with only two false positive detections in one sound file. However, it had the greatest number of false negatives (i.e. poorest recall). The highest performing template had an ROC value of 0.897 and was moderately sensitive but yielded fewer false positives. The third template was excluded. The *L. dumerilii* recogniser comprised four templates from two sites. Three of the templates had very high ROC values (0.87–0.81). The fourth template displayed the greatest number of false positive detections and the poorest ROC value (0.824) was excluded from the recogniser. The *L. peronii* recogniser comprised three templates from a single site. Two templates had the same ROC value of 0.847, and all templates had relatively high precision.

The *C. signifera* recogniser comprised three templates from two sites, two from site S and one from site H (Appendix 1). All three templates performed moderately well, with ROC values between 0.767 and 0.793, modest survey precision and good recall. The *C. parinsignifera* recognisers were constructed from three templates, stemming from two sites. The template from the first site performed poorly, with an ROC value of 0.671. This template detected over 6700 calls in 58 sound files where the species was absent (low precision). The other templates were more precise and performed moderately well, with ROC values around 0.7–0.73.

The *L. tasmaniensis* recognisers performed poorly, with ROC values of 0.562, 0.596 and 0.582. One template performed moderately well for survey recall (0.822) but had poor precision and a very high number of detections in sound files where the species was absent. The other templates performed worse.

Two other species had limited validation data. The *L. raniformis* recogniser comprised three templates from site R. All performed highly, with two templates having no false positives (precision of 1.00), and one template having no false negatives (recall of 1.00). All templates had an ROC of 0.917, however, these performance metrics were calculated from only 24 evaluation sound files and should be interpreted cautiously. The *N. sudelli* recogniser comprised three templates from site M. All templates had a high ROC value of 0.984. All templates had false positive detections in only one file, although the number of detections varied. However, performance was evaluated on only 62 sound files.

### 3.2 Sources of false positive detections

The major sources of false positive detections varied between frog species (Table 3). Insects caused the highest proportions of false positive detections for *C.signifera*, *L. fletcherii* and *C. parinsignifera*. Calls by other frogs also caused many false positives. Other frogs returned the highest false positives for *L.tasmaniensis*, *N. sudelli*, *L. raniformis* and second highest for *C. signifera* and *L. fletcherii*. False positive detections resulting from birds were high for *L. dumerilli*, *L. peronii* and *L. raniformis*. Additionally, weather caused most false positive detections for *L. tasmaniensis*.

**TABLE 3:** False positive detections per category per frog species given as a percentage of total false positive detections of that species.

Sources of false positive detections (%)	*C. signifera*	*L. tasmaniensis*	*L. Fletcherii*	*L. dumerilii*	*L. peronii*	*C. parinsignifera*	*N. sudelli*	*L. raniformis*
Anthropogenic	0.1	9.9	3.3	33.3	0	0	0	13.2
Water sounds	0.2	0.1	0	4.8	7.0	0	0	0
Birds	10.5	0	9.1	52.4	72.1	0	8.2	30.9
Insects	46.5	0	48.1	0	0	66.1	30.8	0
Other geophony	0	0.1	0	0	2.3	33.9	0	1.5
Other animals	0	0	0.4	0	0	0	8.9	0
Other frogs	41.7	50.6	35.3	0	4.6	0	52.1	54.1
Weather	0.8	39.3	3.7	9.5	14.0	0	0	0.2

These false positive detection sources that generated some of the highest error—insects, other frogs and weather—often caused high detections due to their continuous nature—as opposed to just episodic events. These categories of error routinely lasted the entire 5‐min recording, hence resulting in very high false positive detections in a single file. Conversely, false positive detections from sources such as anthropogenic, aquatic, birds, nature or other animals remained relatively low in quantity, as the intervals at which these sounds repeated were infrequent.

## 4 DISCUSSION

Single call annotation, whether manual or via recognisers, is a viable alternative to acoustic indices for monitoring ecological restoration (Linke & Deretic, 2020). While recognisers are commonly treated as one analysis class, there is a gradient in both effort and performance of auto‐detectors. This ranges from largely automated recognisers—typically built‐in software packages such as ‘Kaleidoscope’ (Wildlife Acoustics, 2017)—to completely custom‐built software (Towsey et al., 2012). In all cases, various parameters alter recogniser performance; these may be left as defaults in software or manipulated by the end‐user. Differences in recogniser construction alters performance and this can manifest as poor agreement among recognisers built using different software (Lemen et al., 2015). Relying on recognisers without properly understanding how they operate can be problematic (Russo & Voigt, 2016). In this study, we took a semi‐custom approach; we used a pre‐programmed matching algorithm (Towsey et al., 2012; Ulloa et al., 2016) within the R package monitoR (Katz et al., 2016b), but actively investigated three important parameters that are often overlooked—or, at least, are rarely reported on—in recogniser construction. These parameters were call template selection and representativeness, template construction (including amplitude cut‐off) and the threshold of similarity at which detection is returned (score cut‐off). We argue that there is a need to establish thorough construction and evaluation mechanisms for building recognisers, and for these to be properly reported in the literature.

First, choices pertaining to call template selection are crucial (Katz et al., 2016a; Teixeira et al., 2022). Studies typically report the source of call templates (e.g. whether calls were collected from wild or captive animals), but usually fail to explain the decisions underlying the selection of the exact calls used. For example, were calls free of background noise—and how did this affect recogniser performance? Animal calls exist not in isolation, but within an overall soundscape. As such, representing calls within the context of the soundscapes that we seek to monitor may be important. While our call recognisers perform well overall (Table 2), they are also prone to species‐specific errors. For example, *L. tasmaniensis* recognisers produce false positives for rain events, whereas erroneous detections of *C. parinsignifera* mainly found birds and insects (Table 3).

In this study, we attempted to represent common background noises, such other species' calls and non‐biological sounds (e.g. running water). Although we selected calls that were relatively clear in their structure, we maintained a ‘buffer’ (or a margin) around each selection, in both the time and frequency domains. Since any manual selection of candidate calls will incur a level of human bias, we chose to extract between 100 and 200 templates per species, from which a minimum of 10 were tested and only two or three were chosen for the final recogniser. Although for some rare or cryptic species, call templates can be difficult to acquire, we argue that, as much as possible, recognisers should be built following the testing of many candidate templates.

Another important consideration is the representativeness of species' call types and behaviours (Priyadarshani et al., 2018). For species that exhibit large vocal repertoires, decisions must be made about the call types to feature in recognisers. This should be driven by a program's objectives or research questions; for example, monitoring breeding may require only one or two breeding‐associated call types to feature in the recogniser (Teixeira et al., 2019). Further, geographic variation in call structure (e.g. regional dialects) may also impact recogniser performance and should be investigated when recognisers are intended for use at spatial scales over which call types may vary (Kahl et al., 2021; Lauha et al., 2022; Priyadarshani et al., 2018). If recognisers are used among discrete or isolated populations, call templates may need to represent each area. In this study, we attempted to represent inter‐site variability by selecting candidate call templates from every site where the species was recorded. For several species, the final recognisers comprised templates from more than one site.

Once call templates are chosen, decisions must be made about their construction for use in a recogniser. In binary point matching, call templates are created from a grid of on and off points (i.e. call and non‐call points), which are manipulated by the amplitude cut‐off set by the user (Katz et al., 2016b). In monitoR, the impact of altering amplitude cut‐off can be easily visualised (Figure 2). In this study, we manipulated amplitude cut‐off to show both the call structure and some background noise. Since the recogniser ‘matches’ both the on and off points, finding a suitable balance between these is important. Although visualising and selecting amplitude cut‐off is a manual and somewhat arbitrary process, we considered that the large sample size of candidate templates tested in this study would minimise any bias from this process. However, for studies that test a smaller number of candidate templates, we recommend that each template is tested at several different amplitude cut‐offs.

Finally, an appropriate score cut‐off, which sets the threshold of similarity at which a detection is returned (Figure 3), must be set for each call template. Score cut‐off alters the template's sensitivity and therefore, greatly affects performance. A higher score cut‐off will reduce false positive detections but may increase false negatives (Katz et al., 2016a). Conversely, increasing sensitivity by lowering the score cut‐off will reduce false negatives, but it may reduce precision by returning more false positives. Here, we tested every call template at score cut‐off increments of 0.2 from a low of 3 and measured performance by ROC value. For most species examined, high ROC values indicated that call templates were able to sufficiently trade‐off false positives and false negatives while maximising true positives. This rigorous approach to scoring cut‐off testing allowed us to set highly specific cut‐offs in the final recognisers. However, for species that are rarer or more cryptic, returning sufficient true positives may require a lower score cut‐off with a poorer ROC value. Where detecting most, if not all, calls is important, other performance metrics like recall should be given due consideration. Ultimately, decisions about score cut‐off should be driven by a study's objectives, but we argue that general metrics like ROC values are a good starting point in most cases.

We argue that ecoacoustic researchers and practitioners need to stop treating recognisers like a black box and actively develop, improve and test processes that help evaluation. From the literature, it is currently unclear how reliable recognisers are. Many studies report poor performance (Bohnenstiehl et al., 2018; Gibb et al., 2019; Priyadarshani et al., 2018) but this may be more a function of inappropriate construction, rather than recognition methods per se. Especially recogniser testing is often ignored and recogniser performance is reported by the number of detections in a larger data set. Even when performance is reported, it is often unclear what the source of low recogniser accuracy is. We demonstrated that this could have multiple causes, from badly selected templates to a lack of template calibration, for example amplification or detection cut‐offs. We recommend that recognisers are not treated as static product. They can be refined and adapted as more monitoring data become available. Using this study as an example, we are currently working on a refinement for the recogniser for *L. tasmaniensis* that is based on better template recordings. A complete recommended workflow could start with a recogniser built for a particular species in a particular region, then enhanced by data from other environments, followed by a performance evaluation and refinement as necessary.

### 4.1 Conclusion and future directions

In this study, we have demonstrated the possibility of building high‐quality single‐call recognisers for monitoring ecosystem restoration. These recognisers are now ready to be tested for their response to environmental watering actions. We encourage testing transferability of recognisers to other locations as site specificity of recognisers has rarely been investigated. While commonly used to monitor the outcomes of environmental flows (Gawne et al., 2020; Sarker et al., 2022), amphibians are also not the only soniferous taxonomic group that responds to environmental watering. Birds were responsible for the bulk of the manually annotated response curves described by Linke and Deretic (2020). We strongly encourage additional studies that build call recognisers for water‐dependent birds or alternatively trial the performance of a pre‐built recogniser such as BirdNet (Kahl et al., 2021).

## AUTHOR CONTRIBUTIONS

**Simon Linke:** Conceptualization (equal); data curation (equal); formal analysis (equal); funding acquisition (lead); methodology (equal); validation (supporting); writing – original draft (lead); writing – review and editing (equal). **Daniella Teixeira:** Conceptualization (equal); data curation (equal); formal analysis (equal); funding acquisition (equal); methodology (equal); writing – original draft (supporting); writing – review and editing (supporting). **Katie Turlington:** Conceptualization (supporting); data curation (equal); formal analysis (supporting); investigation (equal); validation (lead); writing – original draft (supporting); writing – review and editing (supporting).

## CONFLICT OF INTEREST STATEMENT

None.

## Supporting information


Appendix S1.

## Data Availability

The documented R workflow can be found in Appendix S1. monitoR recogniser templates can be made available on request, primary sound data are the property of the Forestry Corporation of NSW and needs to be requested directly.
