# Supplementary material for: Evaluating and optimising performance of multi‐species call recognisers for ecoacoustic restoration monitoring
Source: Ecol Evol. 2023 Aug 22;13(8):e10309. doi: 10.1002/ece3.10309 (PMC10443330; doi:10.1002/ece3.10309)
Supplement: Supplementary file 1 — Appendix S1. [file ECE3-13-e10309-s001.zip › ece310309-sup-0001-AppendixS1.docx]

# -----------------------------------------------------------------#

# Step 1: File List Preparation

# -----------------------------------------------------------------#

# Load required libraries

library(monitoR)

library(tuneR)

library(seewave)

# Set the working directory where the WAV files are located

setwd(JCurrentSongmeters_201910SMJ)

# Create a flag variable to indicate if we want to perform full check of files

# Set to 1 to perform full check, 0 to skip the check

fullcheck - 1

# Generate a list of all WAV files in the directory (and subdirectories)

fulllist - list.files(pattern = .wav, recursive = TRUE)

# Convert the list to a data frame

completelist.as.df - as.data.frame(fulllist)

# Loop through each file in the data frame

for(listloop in 1nrow(completelist.as.df)){

# Read the WAV file and extract its properties

detectsamplerate - readWave(fulllist[listloop], header=TRUE)

# Save the properties into the data frame

completelist.as.df$sample.rate[listloop] - detectsamplerate$sample.rate

completelist.as.df$channels[listloop] - detectsamplerate$channels

completelist.as.df$bits[listloop] - detectsamplerate$bits

completelist.as.df$durmin[listloop] - (detectsamplerate[[4]] detectsamplerate[[1]])60

# If fullcheck is set to 1, check for broken bits

if(fullcheck == 1){

fullfile - readWave(fulllist[listloop])

completelist.as.df$buggered.bits[listloop] - length(fullfile@left[fullfile@left == -32768])

}

# Print progress every 50 files

if(round(listloop50) == listloop50){

cat(paste(listloop,....))

}

}

# Save the data frame to a CSV file

write.csv(completelist.as.df,Full List Test.csv)

# -----------------------------------------------------------------#

# Step 2: Recogniser preparation

# -----------------------------------------------------------------#

# Load necessary libraries

library(monitoR)

library(tuneR)

library(RODBC)

library(seewave)

library(lubridate)

library(reshape2)

library(dplyr)

# Clear workspace to free up memory

rm(list = ls()) # remove all objects from workspace

gc(reset = T) # free up memory

# Set working directory where the WAV files are located

setwd("I:\\2021 Recognisers\\Fletcherii\\PosNeg\\NewTemps")

# Define template stack name

templatestack <- "fletchi2021"

stackname <- "fletchi2021"

# Create directory for processed files

dir.create("processed")

# Generate a list of all WAV files in the directory

ref_filelist <- list.files(pattern = ".wav")

NoOfRefcalls <- length(ref_filelist) # count the number of WAV files

# Initialize variables

cutoff_increments <- c(0) # amplification cutoff modifier

refcolnames <- c("amp", "genus", "species", "number", "site", "datetime")

templatenum <- 0

# Open a pdf for plotting

pdf(paste(stackname, ".pdf", sep = ""), width = 4, height = 4)

# Loop over each WAV file to process them

for (callno in 1:NoOfRefcalls) {

# Read the WAV file and extract its properties

refcall_in <- readWave(ref_filelist[callno])

detectsamplerate <- waveHeader(refcall_in)

# Convert to mono if it's stereo

if (detectsamplerate$channels == 2) {

refcall_in <- mono(refcall_in)

}

# Resample to 24kHz if sample rate is different

if (detectsamplerate$sample.rate != 24000) {

refcall_in <- resamp(refcall_in, f = detectsamplerate$sample.rate, g = 24000, output = "Wave")

}

# Delete the original file after reading it

unlink("refcall_in.wav")

# Extract amplitude from the filename

amplist <- strsplit(ref_filelist[callno], '_')

# Create binary point match templates at different amplification levels

for (modifier in cutoff_increments) {

templatenum <- templatenum + 1

amplification <- as.numeric(amplist[[1]][1]) * -1 + modifier

templatename <- paste(amplification, 256, newCols[callno, 4], newCols[callno, 5], sep = "_")

# Create and save binary point match template

bpmtemplate <- makeBinTemplate(refcall_in, wl = 256, frq.lim = c(0.17, 2.5), amp.cutoff = amplification, name = templatename, write.wav = TRUE)

title(paste(templatename))

file.rename("refcall_in.wav", paste("processed\\", templatename, ".wav", sep = "")) # rename and save template files

assign(templatename, bpmtemplate)

bpmlist[templatenum] <- templatename

}

}

# Close the pdf

dev.off()

#===========================CREATE STACK

# Initialize variable to hold binary templates

btemps <- NULL

# Create initial binary template using the first entry in bpmlist

btemps <- combineBinTemplates(get(bpmlist[[1]]))

# Loop through the rest of the bpmlist and add each to btemps

for (callno in 2:templatenum) {

# Get the current binary template from bpmlist

bpmnew <- get(bpmlist[[callno]])

# Combine the current template with the existing templates in btemps

btemps <- combineBinTemplates(btemps, bpmnew)

}

# If you need to change the threshold score cut-off, you can do it as shown below

# The following line is commented out as it's not being used in this script

#templateCutoff(btemps) <- c(9.4, 9.8, 13.4, 8.4, 7.4)

# Combine templates into one stack twice

firsttemps <- btemps

secondtemps <- btemps

btemps <- combineBinTemplates(firsttemps, secondtemps)

# Save the final binary template stack to an RData file

save(btemps, file = paste(templatestack, ".Rdata", sep = ""))

# -----------------------------------------------------------------#

# Step 3: Evaluation loop

# -----------------------------------------------------------------#

# Load necessary libraries

library(ggplot2)

library(monitoR)

library(tuneR)

library(RODBC)

library(seewave)

library(lubridate)

library(reshape2)

library(dplyr)

# Set working directories and create directory for output graphs

setwd("J:\\Experimental")

setwd("I:\\2021 Recognisers\\Fletcherii")

dir.create("Current Run Graphs")

# Define stack name and load respective template file

stackname <- "L_fletcherii_Aug2020"

templatestack <- paste(stackname, ".Rdata", sep = "")

load(templatestack)

# Define bin templates

btemps

# Define bin templates that will be combined later

# (uncomment lines if needed, they are commented as per original script)

# finalstack <- combineBinTemplates(btemps[[3]], btemps[[4]], btemps[[5]])

# finalstack_parasignifera <- combineBinTemplates(btemps[3], btemps[4], btemps[5])

# Save final template stack to file (uncomment lines if needed)

# save(finalstack_parasignifera, file = "Final 3 Temp stack Parasignifera V3.Rdata")

# Read full file list and split into columns for folder, site, data, and filename

fullfilelist <- read.csv("J:\\Current\\2018ListCorrect.csv")

newColNames <- c("folder", "Site","data","filename")

newCols <- colsplit(fullfilelist$fulllist, "/", newColNames)

sm <- songmeter(newCols$filename)

# Combine tables and filter by year

alltable_2019 <- cbind(fullfilelist, newCols, sm)

alltable <- alltable_2019[which(alltable_2019$year == 2018), ]

# Read annotated file list and prepare it for subsetting

sitetable <- read.csv("I:\\Annotated reference calls\\Frog_master datasheet 2018_Allsites.csv")

relative <- melt(sitetable, id.vars = (c("Site","Period","Date")), na.rm = FALSE)

relative[is.na(relative)] <- 0

spec_only <- relative[which(relative$variable == "C..parinsig"), ]

# Subset for presence/absence

spec_only_pres <- subset(spec_only, value != 0)

spec_only_abs <- subset(spec_only, value == 0)

final_list <- rbind(spec_only_pres, spec_only_abs)

# Prepares final list of 0s and 1s for subsetting

final_list$PA <- 0

final_list$PA[final_list$value > 0] <- 1

numpres <- nrow(spec_only_pres)

# Group by PA, sample numpres rows, and arrange by Site

runlist <- final_list %>%

group_by(PA) %>%

sample_n(numpres) %>%

arrange(desc(PA), Site)

# Initialize empty lists and data frames

detectlist <- NULL

templatelist <- NULL

detects <- NULL

fullsumtable <- NULL

summary_to_exp <- NULL

# Create a data frame from the names of the templates

templatelist <- as.data.frame(names(btemps@templates))

templatelist$flag = 1

colnames(templatelist) <- c("template", "flag")

# Loop through each row of the runlist

for(megaloop in 1:nrow(runlist)) {

cat(paste("Run ", megaloop))

# Select the specific row from runlist

chosen_one <- runlist[megaloop,]

# Split the date into day, month, and year

date_selecta <- colsplit(chosen_one$Date, "/", c("day", "month", "year"))

date_selecta$flag <- 1

date_selecta$Site <- chosen_one$Site

# Merge date_selecta and alltable

final <- merge(date_selecta, alltable, by = (c("Site","day", "month", "year")))

# Construct input filename

infilename = paste("J:/Current/", final$fulllist[1], sep = "") # [1] avoids crashes when there are duplicates

# Prepare the recogniser

offset = 0

filename = infilename

# Calculate duration and sampling frequency

detectsamplerate <- readWave(filename, header = TRUE)

dursec <- detectsamplerate[[4]] / detectsamplerate[[1]]

durmin <- as.integer(dursec / 60)

interval = 300 # snippet length in seconds to avoid memory overload

summary <- data.frame() # Create an empty dataframe for every run

# Ensure integer duration in minutes

if (dursec / 60 == as.integer(dursec / 60)){

durmin <- durmin - 1

}

# Cleanup before processing

unlink("snippet_in.wav")

# Match the bin templates to the survey

scores <- binMatch(

survey = filename,

templates = btemps,

time.source = "fileinfo",

write.wav = TRUE,

show.prog = TRUE

)

# Remove temporary file

unlink("snippet_in.wav")

# Convert scores to peaks

pks <- findPeaks(score.obj = scores)

# Extract the detections

detects <- getDetections(pks)

# Compute summary stats if there are detections

if (nrow(detects) > 0) {

means_by_rec <- aggregate(detects$score, by = list(detects$template), FUN = mean)

n_by_rec <- aggregate(detects$score, by = list(detects$template), FUN = length)

sd_by_rec <- aggregate(detects$score, by = list(detects$template), FUN = sd)

max_by_rec <- aggregate(detects$score, by = list(detects$template), FUN = max)

sumtable <- cbind(n_by_rec, means_by_rec[,2], sd_by_rec[,2], max_by_rec[,2])

colnames(sumtable) <- c("template", "Num_Detecs", "Mean Score", "SD Score", "Max Score")

fullsumtable <- merge(sumtable, templatelist, by = "template", all = TRUE)

fullsumtable$presence <- chosen_one$value

fullsumtable$filename <- final$filename[1]

fullsumtable$filenumber <- megaloop

colnames(fullsumtable) <- c("template", "Num_Detecs", "Mean Score", "SD Score", "Max Score", "flag", "Presence", "Filename", "File_no")

summary_to_exp <- rbind(fullsumtable, summary_to_exp)

outfilename <- paste("Current Run Graphs/", final$filename[1], "_detects.csv", sep = "")

write.csv(detects, outfilename)

detects$presence <- chosen_one$value

detects$filename <- final$filename[1]

detectlist <- rbind(detectlist, detects)

}

}

# Handling cases when no detections were made

if (nrow(detects) < 1){

sumtable <- templatelist

sumtable <- cbind(sumtable, NA, 0, 0, 0)

sumtable$presence <- chosen_one$value

sumtable$filename <- final$filename[1]

sumtable$filenumber <- megaloop

colnames(sumtable) <- c("template","flag", "Num_Detecs", "Mean Score", "SD Score", "Max Score", "Presence", "Filename", "File_no")

fullsumtable <- sumtable

summary_to_exp <- rbind(fullsumtable, summary_to_exp)

detects <- data.frame(

template = NA,

date.time = NA,

time = 0,

score = 0,

presence = chosen_one$value,

filename = final$filename[1]

)

detectlist <- rbind(detectlist, detects)

}

# Plotting detected peaks

soundfilename <- paste("Current Run Graphs/", chosen_one$value, " ", final$filename, sep = "")

png_filename <- paste(soundfilename, ".png", sep = "")

png(png_filename, width = 8000, height = 600)

plot(pks, ask = FALSE, t.each = 300)

dev.off()

# Writing summary and detection data to CSV

write.csv(summary_to_exp, paste(stackname, ".csv", sep = ""))

write.csv(detectlist, paste("detectlist ", stackname, ".csv", sep = ""))

# For Demo

stackname <- "C_parinsignifera_V3"

detectlist <- read.csv(paste("detectlist ", stackname, ".csv", sep = ""))

detectlist_no0 <- filter(detectlist, score != 0)

summary_to_exp <- read.csv(paste(stackname, ".csv", sep = ""))

# Standardize down presence values >0

detectlist$rocPA <- ifelse(detectlist$presence > 0, 1, 0)

# ROC Analysis

roctable <- NULL

summarised <- NULL

toexp <- NULL

thresh <- 8

nosites <- length(unique(detectlist$filename))

nosites <- length(unique(summary_to_exp$Filename))

detectlist_nulls <- filter(detectlist, score == 0)

detectlist_no0 <- filter(detectlist, score != 0)

summary_to_exp_nulls <- summary_to_exp %>% filter(is.na(Num_Detecs)) %>% filter(Presence == 0)

list_of_templates <- as.data.frame(unique(detectlist$template))

# More ROC Analysis...

summary_to_exp_nullstats <- summary_to_exp_nulls %>% group_by(template) %>% summarize(addedcorr = n())

thresh <- 7

for (thresh in seq(3, 20, 0.2)) {

# Perform several calculations here

# ...

}

# Saving ROC table and plotting ROC

write.csv(roctable, paste(stackname, "roctable.csv"))

roctable <- read.csv("roctable.csv")

ggplot(roctable, aes(cutoff, roc)) + geom_point() + facet_wrap(~ template)

# Aggregating ROC and saving

aggregator <- roctable %>% group_by(template) %>% slice(which.max(roc))

write.csv(aggregator, paste(stackname, "aggregated.csv"))

# Template cutoffs, saving and combining bin templates

templateCutoff(btemps) <- aggregator$cutoff

save(btemps, file = paste(stackname, "_Correct_Cutoffs.Rdata", sep = ""))

runnertemps <- combineBinTemplates(btemps[15], btemps[5], btemps[18], btemps[20], btemps[9], btemps[22])

templateCutoff(runnertemps) <- c(4.4, 4.8, 4, 8, 5, 5.4)

save(runnertemps, file = paste(stackname, "_For_Run.Rdata", sep = ""))

# Saving final runners

finalrunners_peronii <- combineBinTemplates(runnertemps[2], runnertemps[3], runnertemps[5])

save(finalrunners_peronii, file = paste(stackname, "_Final_For_Run.Rdata", sep = ""))
